# Side Chain Hydrophobicity Modulates Therapeutic Activity and Membrane Selectivity of Antimicrobial Peptide Mastoparan-X

**DOI:** 10.1371/journal.pone.0091007

**Published:** 2014-03-12

**Authors:** Jonas R. Henriksen, Thomas Etzerodt, Torben Gjetting, Thomas L. Andresen

**Affiliations:** 1 DTU Chemistry, Department of Chemistry, Technical University of Denmark, Center for Nanomedicine and Theranostics, Kongens Lyngby, Denmark; 2 DTU Nanotech, Department of Micro- and Nanotechnology, Technical University of Denmark, Center for Nanomedicine and Theranostics, Kongens Lyngby, Denmark; Centro Nacional de Biotecnologia - CSIC, Spain

## Abstract

The discovery of new anti-infective compounds is stagnating and multi-resistant bacteria continue to emerge, threatening to end the “antibiotic era”. Antimicrobial peptides (AMPs) and lipo-peptides such as daptomycin offer themselves as a new potential class of antibiotics; however, further optimization is needed if AMPs are to find broad use as antibiotics. In the present work, eight analogues of mastoparan-X (MPX) were investigated, having side chain modifications in position 1, 8 and 14 to modulate peptide hydrophobicity. The self-association properties of the peptides were characterized, and the peptide-membrane interactions in model membranes were compared with the bactericidal and haemolytic properties. Alanine substitution at position 1 and 14 resulted in higher target selectivity (red blood cells versus bacteria), but also decreased bactericidal potency. For these analogues, the gain in target selectivity correlated to biophysical parameters showing an increased effective charge and reduction in the partitioning coefficient for membrane insertion. Introduction of an unnatural amino acid, with an octyl side chain by amino acid substitution, at positions 1, 8 and 14 resulted in increased bactericidal potency at the expense of radically reduced membrane target selectivity. Overall, optimized membrane selectivity or bactericidal potency was achieved by changes in side chain hydrophobicity of MPX. However, enhanced potency was achieved at the expense of selectivity and vice versa in all cases.

## Introduction

Antibiotics are one of the most important inventions in biomedical research, yearly saving the lives of millions of people. With the discovery of penicillin by Alexander Fleming in 1928, the “antibiotic era” and research in antibiotics was initiated, which led to the invention of drugs like methicillin, vancomycin, linezolid, and the lipo-peptide daptomycin [1]. However, the wide use of anti-infective drugs in hospitals and industrial farming has spawned the emergence of multi-drug resistant bacterial strains [2], and the demand for new and more potent drugs is imperative [1].

Antimicrobial peptides (AMPs) offer themselves as potential drugs for treating bacterial, fungal, and viral infections, and thus as a new class of antibiotics. In higher eukaryotes such as plants and vertebrates, antimicrobial peptides are a natural part of the innate immune system, and serve as the first line of defence against infections [3]. In humans, these peptides are primarily located on skin and on mucosal surfaces, where they protect e.g. the oral tract, lungs, and intestines against bacterial and fungal infections. Important human AMP classes are the defensins, cathelicidins, and histatins [4]. These small molecule drugs have been optimized though evolution to combat invading microorganisms, and several have been shown to exhibit activity towards various bacteria, viruses, fungi, and parasites [5].

AMPs are cationic amphipathic molecules with a typical size of 10–40 amino acids, which carry a net positive charge (typically 1–10 charged residues). The sequence of the peptide is comprised of hydrophilic and hydrophobic amino acid residues, rendering the peptide soluble in both aqueous and lipid phases. In water, the peptide is largely unstructured. Upon insertion into lipid membranes, the peptide adopts a secondary structure, and displaces the hydrophobic and hydrophilic residues of the peptide onto two different faces, which facilitate the insertion into the membrane [6]. Typical secondary structures attained upon membrane insertion are the beta-sheet and alpha-helical peptide conformations. Once inserted into the membrane, the mode of action differs between peptides; some translocate across the membrane [7], [8], some associate into clusters [9] while others form pores on the membrane [10], [11]. Both peptide clusters and pores are heterogeneous structures that permeabilize the membrane. The advantages of AMPs over conventional antibiotics are numerous; they exhibit broad-spectrum activity, they operate via non-receptor mediated mechanisms, and most importantly, they rarely induce resistance [12]. However, AMPs have not yet reached their potential as novel antibiotics, and further exploration of these compounds is needed.

From a biophysical point of view, the interaction of AMPs with various types of lipid membranes has been well characterized, and it is argued in the literature that the peptides' ability to select between mammalian and bacterial membranes is governed by electrostatic interactions between the AMP and the target membrane [13], [14], [15]. Moreover, formation of secondary structure has been shown to be a driver for peptide insertion into membranes [16]. Based on this knowledge, several strategies for optimization of AMPs potency have been investigated including: (i) Increase of peptide charge [17], (ii) modulation of hydrophobicity and hydrophobic moment [18], [19] and (iii) stabilization/destabilization of the peptide secondary structure [20], [21]. However, balancing peptide hydrophobicity and hydrophilicity is non-trivial, which renders the optimization process far from straight forward.

The present work, focus on enhancing the understanding of how changes in peptide hydrophobicity, via acylation or alkylation, impacts selectivity and potency of the peptide. Within this line of optimization, acylation of potent midsized AMPs (15–30 AA) [22], [23], [24] and ultra short tetra and penta peptides [25], [26] have been investigated among others. In most studies, a N-terminal conjugation strategy is utilized because of simplicity. This approach has resulted in peptides with higher and broader activity spectrum; however, the activity has been achieved at the expense of membrane/target selectivity leading to more haemolytic analogues [26], [27]. The majority of these studies emphasize bactericidal potency of the peptide as their key optimization parameter and pay less attention to haemolytic properties, mode of membrane action, aggregation and folding etc. As a consequence, the selected peptide analogues are often more haemolytic than the native peptides, which hampers their use. Therefore, many studies of C4–C18 acylated peptides point to the conclusion that C12–C14 constitutes the optimal acyl chain length range [22], [26]. However, peptide selectivity is tightly connected to the balance of hydrophilic and hydrophobic residues. Therefore, the proper acyl/akyl chain length to use (if any) depends on the hydrophobicity of the native peptide as well as the position of alkylation/acylation on the peptide backbone.

**Figure 1:**
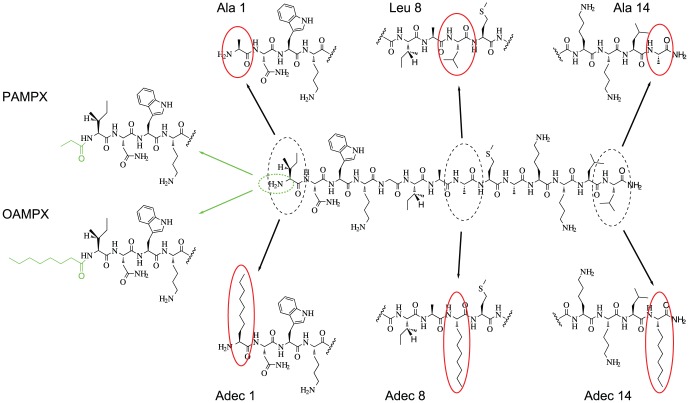
Structures of MPX and eight analogues. Ala1 and Ala14 have alanine substitution at position 1 or 14, and are analogues with reduced hydrophobicity. Leu8 has a leucine substitution in position 8 resulting in augmented hydrophobicity. Adec1, Adec8 and Adec14 have 2-amino-decanoic acid substitution in position 1, 8 or 14 respectively, and constitute the most hydrophobic analogues of MPX considered in this study. PAMPX and OAMPX are N^α^-terminal propanoic and octanoic acid acyl analogues of MPX, respectively.

In a recent work, we discovered that membrane selectivity and affinity of mastoparan X (MPX) were enhanced by short-chain acylation. In addition, selectivity was abolished by medium-chain acylation, possibly rendering the investigated octanoic acid analogue inefficient in targeting bacteria over mammalian cells [14]. Moreover, we hypothesized that the observed changes in membrane selectivity and affinity were linked to the small proximity of the acylation point at the N-terminal and neighbouring charges. In the present study, we expand on these observations via an extended set of MPX analogues containing six new analogues. The full set of peptides is characterized using biophysical methods and is screened for haemolytic and bactericidal activity. Native MPX (H-INWKGIAAMAKKLL-NH2) is a 14 amino acid peptide with a net positive charge of +4. MPX belongs to the mastoparan family of antimicrobial peptides, and is found in high concentration in wasp venom [27]. MPX displays considerable antimicrobial activity against Gram-positive bacteria; however, MPX also shows substantial haemolytic activity. The current set of peptides contains eight synthesized MPX analogues as shown in Fig. 1. Ala1 and Ala14 have an alanine substitution at position 1 or 14, Leu8 has a leucine substitution at position 8, Adec1, Adec8 and Adec14 have a 2-amino-decanoic acid substitution at position 1, 8 or 14, respectively. PAMPX and OAMPX are the N^α^-terminal propanoic and octanoic acid acyl analogues of MPX, respectively, which were investigated previously [14]. The new analogues (Ala1, Ala14, Leu8, Adec1, Adec8 and Adec14) are designed to have varying hydrophobicity, at position 1, 8 and 14 of the MPX sequence, while keeping the peptide charge constant; a condition that is not met by the N-terminal acylated analogues PAMPX and OAMPX. Thus, the current set of peptides contain analogues of constant nominal charge, with either an Ala, Leu (or Ile) or 2-amino-decanoic acid substitutions at each position (1, 8 or 14), which provides a side-chain size-range from C1 to C4 to C8.

In order to reveal effects related to changes in side-chain hydrophobicity and/or peptide backbone alkylation position, these peptide analogues were screened for their mode of membrane interaction, tendency to aggregate, membrane partitioning and haemolytic and bactericidal properties.

## Materials and Methods

### Materials

All Fmoc (9H-fluoren-9-yl-methyloxycarbonyl) L-amino acids and HATU (1-[Bis(dimethylamino)methylene]-1*H*-1,2,3-triazolo[4,5-*b*]pyridinium 3-oxid hexafluorophosphate, *N*-[(Dimethylamino)-1*H*-1,2,3-triazolo-[4,5-*b*]pyridin-1-ylmethylene]-*N*-methylmethanaminium hexafluorophosphate *N*-oxide) were purchased from GLS China (GL Biochem, Shanghai, China). Fmoc-2-amino-decanoic acid was obtained from Watanabe Chemical Industries, Ltd (Hiroshima, Japan). Lys and Trp were side-chain-protected as tert-butoxycarbonyl, Asn was sidechain protected with trityl. Tentagel resin was purchased from NovaBiochem (Merck, Darmstadt, Germany). TFA (trifluoroacetic acid), TIS (triisopropylsilane), HEPES (N-(2-hydroxyethyl)piperazine-N-(2-ethanesulfonic acid)), 2,4,6-collidine and pyrene were purchased from Sigma-Aldrich (Brøndby, Denmark). Pyridine, sodium hydroxide pellets and sodium chloride was purchased from J.T. Baker Chemicals (Phillipsburg, NJ, USA). Filter supports and Hamilton extrusion syringes, POPG (1-palmitoyl-2-oleoyl-sn-glycero-3-[phospho-rac-(1-glycerol)] sodium salt), and POPC (1-palmitoyl-2-oleoyl-sn-glycero-3-phosphocholine) were purchased from Avanti Polar Lipids (Alabaster, AL). All compounds were used without further purification. Membrane filters for extrusion (100 nm pore size, polycarbonate) were purchased from Whatman (Maidstone, UK). Cuvettes (4×10 mm, quartz, Suprasil) were purchased from Hellma (Müllheim, Germany). Purified human erythrocytes for the haemolysis studies were provided by Bioneer (Hørsholm, Denmark), and were stored at 4°C for a maximum of three days before use.

### Peptide synthesis and purification

Peptides were prepared by manual Fmoc solid phase peptide synthesis. Briefly, 3 equivalents of Fmoc-amino acid, 2.98 eq. of HATU and 6 eq. of 2,4,6-collidine dissolved in DMF were used for each coupling. Gly required systematic double coupling. Coupling of each amino acid and Fmoc deprotection was monitored using the ninhydrin test. Occasionally, a small portion of resin was cleaved, and the peptide fragment identity was verified by mass spectrometry to ensure full conversion in the individual synthetic steps. The peptides were cleaved from the resin using a mixture of TFA∶TIS∶H_2_O (95∶2.5∶2.5), and the volume of the cleavage mixture was reduced to ∼5% in vacuum or until slight precipitation occurred. Water was added and the resulting slurry was subsequently lyophilized. The lyophilized crude peptides were purified by preparative HPLC and analysed by analytical HPLC and mass spectrometry.

Preparative HPLC was conducted using a Waters 2489 system equipped with a Waters Xterra RP18 OBD column, 19×150 mm, 5 µm particle size. Analytical HPLC was conducted using a Shimadzu LC-2010C system equipped with a Waters Xterra RP18 OBD column, 4.6×150 mm, 5 µm particle size. The peptide was detected by UVvis measured at λ = 206 nm and λ = 280 nm for both preparative and analytical HPLC. Identity of the peptide was verified by electrospray ionization mass spectrometry (ESI) using an Agilent 1100 LC-MSD with automated external syringe injection (flow rate 5 µl/min.). The ESI spectra were obtained in negative mode by scanning in the range from m/z 300–2000.

### Peptide stock solutions

Peptide stock solutions were prepared in a buffer containing 10 mM HEPES and 100 mM sodium chloride (pH = 7.4). All peptide concentration were determined by UV-VIS (Shimadzu UV-1700 PharmaSpec) using the molar extinction coefficient of tryptophan (ε_tryptophan,280 nm_ = 5600 cm^−1^ mol^−1^) [28].

### LUV preparation

Lipid stock solutions of POPC and POPG were prepared in chloroform∶methanol (9∶1). Mixtures of POPC∶POPG (3∶1) were prepared and the organic solvent was evaporated under a gentle stream of nitrogen. The lipid films were kept in vacuum (P<0.1 Pa) overnight in order to remove the residual solvent. Buffer containing 10 mM HEPES and 100 mM sodium chloride (pH 7.4) was added to the lipid films, and they were hydrated for 60 min followed by extrusion through 100 nm using an Avanti mini-extruder. Dynamic Light Scattering (DLS, Brookhaven Instrument Corporation, ZetaPALS) was used for measuring the liposome size distribution. The effective lipid concentration was determined by inductively coupled plasma atomic emission spectroscopy (ICP-AES Vista AX, Varian).

### Pyrene-cmc assay

Fluorescence excitation spectra were collected using an Olis modernized SLM8000 fluorescence spectrophotometer equipped with single-grating emission and double-grating excitation monochromator. The fluorometer was equipped with photon counter detectors. Pyrene was used to probe micellation as it is sensitive to environment hydrophobicity. Peptides were incubated in concentrations of 0.1–1000 µM in 10 mM HEPES, 100 mM sodium chloride, pH 7.4 buffer containing 75 nM pyrene. Samples were incubated for 2 h at 60°C followed by incubation overnight at room temperature and kept in the dark until measured. Measurements were carried out at 37°C. Excitation spectra were collected using emission wavelength 390 nm and 16 nm emission slit sizes. Excitation spectra were scanned in range 310–350 nm with excitation slits sizes of 0.5 nm [29]. The excitation spectrum shift denotes the formation of micelles/aggregates (hydrophobic cavity), which is gauged by intensity ratio *R = I_339_/I_333_* [29],[30]. The plot of *R* versus the detergent/peptide concentration, c, measures the partitioning of pyrene into the formed micelles/aggregates. The intensity ratio is fitted using the sigmoidal function 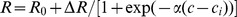, where *R_0_* and *ΔR* denote the initial and change in intensity ratio, respectively. *α* and *c_i_* denote the normalized slope and peptide concentration at which curve inflection occurs, respectively. In the present work, cmc is determined as the onset of the sigmoidal curve, which we have defined via the criteria 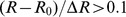 and hence 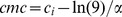. The slope of the sigmoidal curve reflects the cooperativity of the micelle/aggregate formation process and the partitioning properties of pyrene into the given aggregate. The experiment was conducted in triplicates.

### Peptide partitioning by ITC

The partitioning of AMPs onto lipid membranes was investigated by isothermal titration calorimetry (ITC, iTC200, Microcal, 204 µL cell volume). For the peptide analogues MPX, Ala1 and Ala14, 10 mM POPC∶POPC (3∶1) LUVs were injected (19×2 µL) into a 20 µM peptide solution at T = 37°C. The speed of the stirrer was 1000 rpms, and all titration were initiated with a 0.5 µL pre-injection. The heat-traces were integrated using custom made software [31], and the heat of partitioning was analysed using a partitioning isotherm based on Gouy-Chapman-Stern description of the electrostatic interaction of the peptide and membrane [14], [32]. The effective charge of the peptide (*z_eff_*), the partitioning coefficient of insertion (*K_ins_*), the molar enthalpy of partitioning (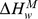), and the heat of dilution (*q_dil_*) were extracted as fitting parameters. The effective partitioning coefficient was derived as 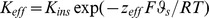 where *F* is Faradays constant, 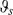 is the electrostatic potential evaluated at the interface of a fully charged membrane (25mol% negative charge before insertion of the first peptide), and *R* is the ideal gas constant. The instrument was electrically calibrated, and blank titration of LUVs into buffer was performed to ensure that heat of dilution could be modelled as a constant in the data analysis. Each titration was conducted in triplicates.

### Mode of peptide-membrane interaction gauged by ITC

The nature of the peptides' membrane interaction was studied as a function of lipid/peptide ratio (L/P) by ITC (iTC200, Microcal). All experiments were conducted by injection of 20 mM LUVs into 100 µM peptide solution. For OAMPX, 8 mM LUVs were used. The experiments were conducted as 38×1 µL injections, T = 37°C, and the speed of the stirrer was 1000 rpms. All titrations were conducted in triplicates.

### Haemolysis assay

The assay was conducted as described in Etzerodt et al. [14].

### Determination of minimal inhibitory concentration (MIC)

A stock dilution series with ten points, ranging from 1 µM to 1 mM, was prepared in sterile PBS from freshly dissolved peptides. The dilution series was dispensed in triplicates in 96-deepwell microtiter plates (VWR Denmark, Herlev, Denmark), with 15 µl in each well. Multidispensing with electronic pipettes (Biohit, Tåstrup, Denmark) was used throughout. *Lactococcus lactis*, strain MG1363 (ATCC, LGC Standards AB, Boras, Sweden) and *Escherichia coli*, strain INV-α (Invitrogen, Nærum, Denmark) were grown in M9 (Sigma Aldrich, Brøndby, Denmark) and LB medium (Difco, BD Biosciences, Brøndby Denmark), respectively. A single colony was grown overnight at 30°C and 37°C for *L.lactis and E.coli respectively*. The next day, a fresh culture was started with a twenty-fold dilution of the cells and grown for exactly 60 min (optical density was approximately 0.4 at 570 nm). The cell culture was diluted (300 fold for *L.lactis* and 1000 fold for *E.coli*) before adding 150 µl to microtiter plates already containing 15 µl peptide solution and 135 µl fresh medium. Hence, the final concentration of the peptide dilution series ranged from 0.05 µM to 50 µM. Plates with *L.lactis* were sealed with lid and parafilm, and plates with *E.coli* were sealed with a gas-permeable tape (VWR Denmark, Herlev, Denmark). The plates were incubated at the respective temperatures for 20 hours with shaking (150 rpm). For each measurement, 100 µl of the cell culture was transferred to clear, flat-bottomed microtiter plates, and the absorbance at 570 nm was determined using a Victor3 plate reader (Perkin Elmer, Skovlunde, Denmark). The measurement was conducted in triplicates.

## Results and Discussion

### Aggregation

**Figure 2:**
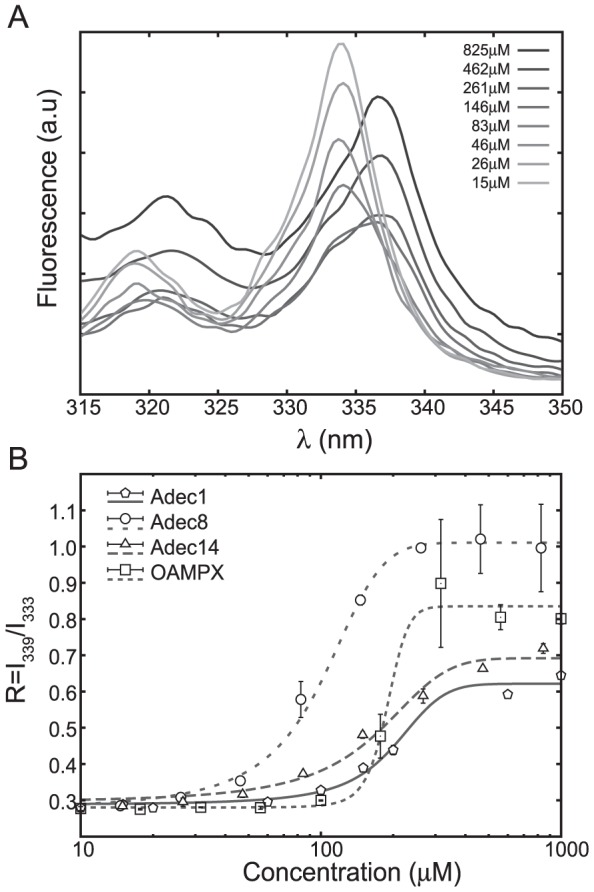
Critical micelle concentration assessed via fluorescence spectroscopy. As an example, pyrene fluorescence excitation spectra are shown for a concentration sequence of Adec8 in (A). The formation of micelles is monitored via partitioning of pyrene into the hydrophobic cavity of the forming micelles. This induces the observed peak-shift of pyrene (A), which is quantified via the intensity ratio R = I_339_/I_333_ plotted in (B) as a function of peptide concentration. In the current work, the cmc is defined as the point where R deviates from the background signal.

The cmc values for MPX and analogues, which were investigated using pyrene as a probe for micelle/aggregate formation (see Fig. 1), are given in Table 1. No shift in the pyrene excitation spectrum (Fig. 2A) was observed for MPX, Ala1, Ala14 and Leu8 in the covered concentration range, and hence cmc >1 mM for these analogues. For the C8 modified analogues, cmc followed the sequence OAMPX>Adec1>Adec8∼Adec14. For simple non-ionic detergents, the cmc is governed by the size of the hydrophobic tail while the size of the hydrophilic head has smaller impact [33]. Therefore, if we consider the MPX analogues as simple detergents, the Adec and OA analogues are expected to have similar tendency to aggregate with minor variations reflecting the acylation/alkylation position and charge of the peptide. Indeed, the Adec1, Adec8, Adec14 and OAMPX analogues are found to have similar cmc values in correspondence with simple detergent theory. However, a slight increase in cmc is observed as the position of the acylchain substitution is moved from the C towards the N terminal. Based on the Wimley and White hydrophobicity scale [34], Adec8 is expected to be more hydrophobic than Adec1, which is predicted to be more hydrophobic than Adec14. This prediction correlates to the observed cmc sequence Adec1>Adec8, but not to Adec1>Adec14. OAMPX, however, has a lower nominal charge than the Adec analogues, which in most cases translate to lower cmc values, contrary to the observed trend. In addition, larger aggregates visible to the eye were observed for Adec8 at concentrations above 125 µM. These variations in cmc, and the aggregation phenomena of Adec8, may also be explained by differences in flexibility of the peptide, which possibly adopts different secondary structures depending on the constraints imposed by the hydrophobic chain. For Adec8, which is alkylated at the midpoint of the peptide sequence, the peptide may serve as a bulky headgroup on top of the C8 acyl chain, which could cause formation of different structures than the ones formed by the linear molecular constructs like OAMPX, Adec1 and Adec14. In studies by Tirrell and coworkers, C14 acylation of the peptide AKK (YGAAKKAAKAAKKAAKAA) resulted in a cmc of 130 µM [22]. The Adec1, Adec8, Adec14 and OAMPX analogues investigated in the present work thus have equivalent cmc values as the C14 AKK analogue reflecting the larger hydrophobicity of MPX compared to AKK.

**Table 1:** Compilation of results.

	*cmc*	*z_eff_*	*K_ins_/10^5^*	*K_eff_/10^8^*	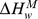	*MIC* (*L. lactis*)	*MIC* (*E. coli*)	*H5*
MPX	>1000	2.5±0.1	4.2±0.2	1.6±0.2	−24.0±0.3	2.5±0.5	8±3	18±2
Ala1	>1000	4.3±0.1	1.1±0.1	25±7	−18.4±0.3	18±7	33±8	230±10
Ala14	>1000	2.9±0.1	1.4±0.3	1.1±0.2	−16.2±0.3	21±4	33±8	220±20
PAMPX	>1000	**-----------**	**-----------**	**-----------**	**-----------**	3.3±0.8	21±4	25±1
Leu8	>1000	**-----------**	**-----------**	**-----------**	**-----------**	2.5±1.3	7±3	14±2
Adec1	90±10	**-----------**	**-----------**	**-----------**	**-----------**	1.7±0.4	3.3±0.8	0.7±0.1
Adec8	30±7	**-----------**	**-----------**	**-----------**	**-----------**	4.2±0.8	4.2±0.8	1±1
Adec14	10±20	**-----------**	**-----------**	**-----------**	**-----------**	1.7±0.4	3.3±0.8	0.8±0.2
OAMPX	150±50	**-----------**	**-----------**	**-----------**	**-----------**	3.3±0.8	5±1	1.1±0.3

Critical micelle concentration (*cmc* in µM units), effective charge (*z_eff_*) of the peptide, partition coefficient of insertion (*K_ins_*), effective partition coefficient (*K_eff_*), molar enthalpy of partitioning (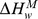 in kJ/mol units), minimal inhibitory concentration (*MIC* in µM units) and the haemolytic potency is given as the concentration *H5* (in µM units) at which 5% haemolysis has been obtained. Membrane partitioning data for PAMPX, Leu8, Adec1, Adec8, Adec14 and OAMPX could not be analysed and these cells are marked with a dashed line.

### Membrane partitioning by ITC

**Figure 3:**
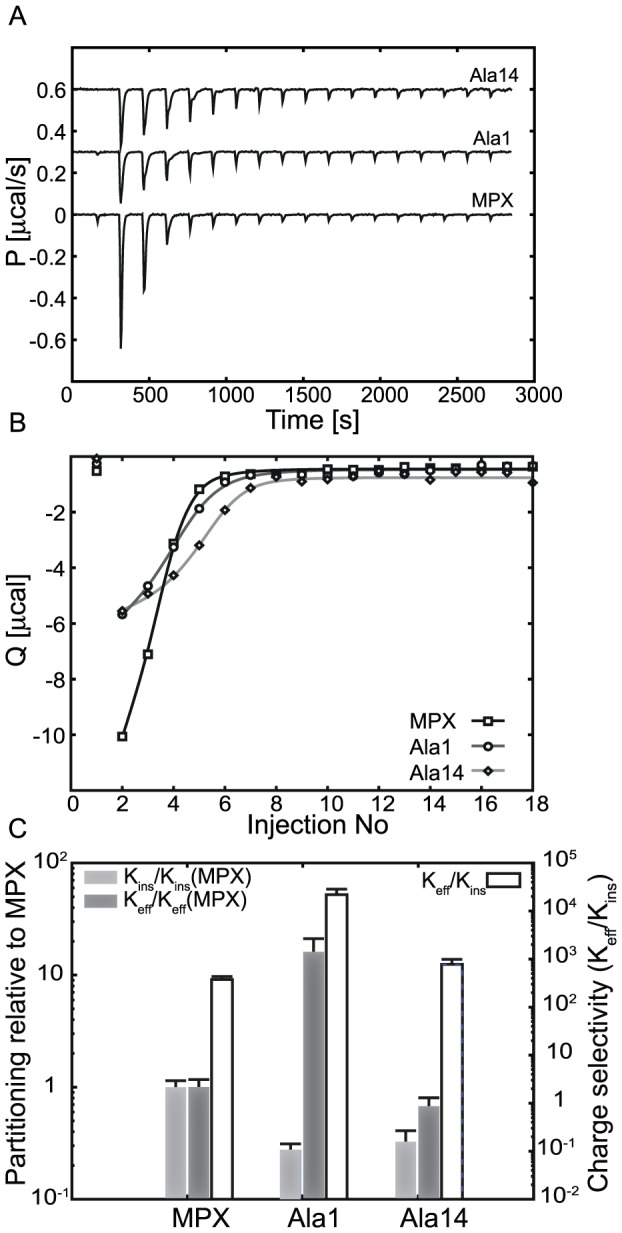
Partitioning of MPX, Ala1 and Ala14 onto POPC∶POPG (3∶1) LUVs studied via ITC at 37°C. In panel (A), heat traces of 25 mM LUVs injected into 20 µM peptide (19×2 µL injections) are shown. Panel (B) shows the corresponding heat of reaction, Q, as a function of number of injections. The solid lines in (B) represent fits to the data. In panel C, the change in potency of Ala1 and Ala14 for neutral or anionic lipid membranes measured relative to MPX, is evaluated via the ratio *K_ins_*/*K_ins_(MPX)* and *K_eff_/K_eff_(MPX)* respectively. The membrane charge selectivity of the peptide (ability to select between neutral and anionic lipid membranes) is assessed via the partitioning coefficient ratio *K_eff_/K_ins_* shown in (C).

For all analogues, partitioning into a POPC∶POPG (3∶1) lipid membrane was investigated by ITC. Heat-traces and corresponding heat of reaction are shown in Fig. 3 A–B for the titration of 10 mM POPC∶POPG (3∶1) 100 nm LUV's into 20 µM peptide. PAMPX, Leu8, Adec1, Adec8, Adec14 and OAMPX exhibited complex heat-traces on ITC, which hampered ITC based analysis of these analogues. This behaviour is attributed to secondary processes such as pore formation, lipid segregation or membrane solubilisation/micellation. The ITC results for or MPX, Ala1 and Ala14, which are summarized in Table 1, are interpreted using a partitioning isotherm based on the Gouy-Chapman-Stern model [14], [32]. Here *K_0_* quantifies the interaction of the analogue with a neutral/zwitterionic membrane; *K_eff_*, measures interaction of the analogue with a negatively charged membrane, and the effective charge, *z_eff_*, governs the electrostatic interactions of the lipid membrane and the peptide. The partitioning process is exothermic for Ala1, Ala14, and MPX, and the molar enthalpy of partitioning (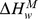) increases upon alanine substitution for both Ala1 and Ala14. For MPX, the partitioning coefficients determined by ITC (Table 1) agree with our previous result (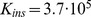 and 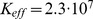), which was obtained via tryptophan fluorescence measurements [14]. However, the effective charge obtained by ITC (Table 1), is slightly larger than our previous result of 1.7 [14], which accounts for the increase in *K_eff_* in our current measurement. Schwartz and co-workers found a partitioning coefficient of insertion (MPX into POPC LUVs), 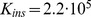 [35], which is in accordance with our result.

Overall, Ala1 and Ala14 show reduced *K_ins_* and increased *z_eff_* and 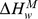 as a result of alanine substitution. The observed inverse correlation of *z_eff_* and *K_ins_* is in accordance with previous findings by White and co-workers, who showed that the effective charge is inversely proportional to the free energy of inserting a peptide into a lipid membrane [36]. The alanine substituted analogues, Ala1 and Ala14, are less hydrophobic compared to MPX, which translates to a reduction in the partitioning coefficient of insertion as expected. For Ala1, the reduction in *K_ins_* is compensated by an augmented effective charge (compared to MPX), which leads to an overall increase of the effective partitioning coefficient (*K_eff_*) when compared to MPX. For Ala14, the increase in effective charge does not balance the decrease in *K_ins_* yielding an overall reduction of *K_eff_*. In order to evaluate the performance of the peptide analogues, the membrane charge selectivity (ability to distinguish charged and neutral lipid membranes) is assessed via the ratio 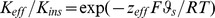 (Fig. 3C). The relative change in potency (propensity for insertion/partitioning) compared to MPX is evaluated via the ratio *K_eff_/K_eff_(MPX)* or *K_ins_/K_ins_(MPX)* for anionic and neutral lipid membranes, respectively (Fig. 3C). Based on this analysis, Ala1 shows increased charge selectivity, increased potency towards anionic lipid membranes, and reduced potency towards neutral lipid membranes when compared to MPX (Fig. 3C). Ala14 displays a minor increase in charge selectivity, but a reduction in potency towards both anionic and neutral lipid membranes compared to MPX (Fig. 3C). Based on the ITC results, we predict Ala1 to be more selective, more potent towards bacteria, and less haemolytic than MPX. Ala14 however, is expected to be less potent and haemolytic, but slightly more selective than MPX. The observed increase in the *K_eff_/K_ins_* ratio for the Ala analogues (reduced hydrophobicity compared to MPX) is also in line with our previous finding for OAMPX. In that study, we found that *K_eff_/K_ins_* was reduced in response to an increased hydrophobicity [14].

### Mode of peptide-membrane interaction gauged by ITC

**Figure 4:**
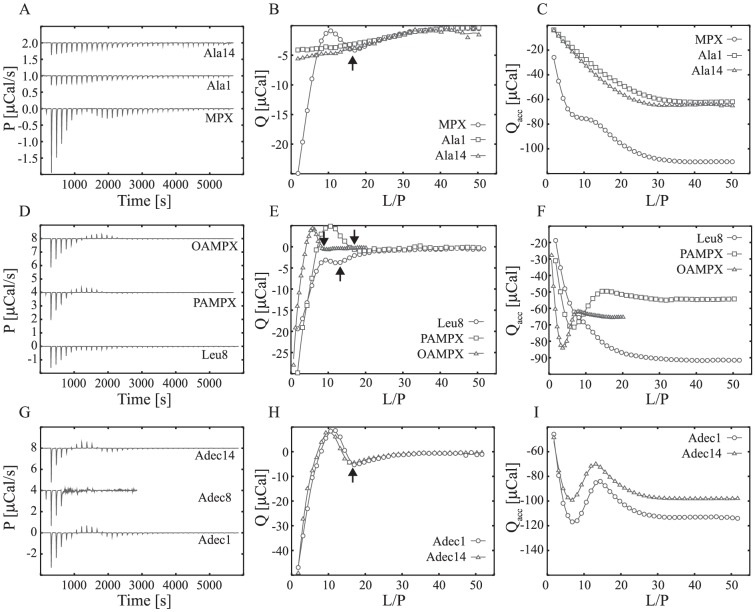
Mode of peptide-membrane interaction evaluated using ITC at 37°C. Panel A–C shows the heat trace of 20 mM POPC∶POPG (3∶1) LUVs injected into 100 µM peptide (38×1 µL injections), except for OAMPX which was titrated using 8 mM LUVs. The corresponding heat of reaction (Q) and accumulated heat of reaction (Q_acc_) are shown in panel D–F and G–I respectively. The arrows in panel D–F highlight the endpoint of the secondary process and the solid lines serve to guide the eye.

The mode of peptide-membrane interaction was investigated by ITC. This study was conducted using a modified titration protocol having an increased peptide concentration (in the ITC cell) compared to the partitioning studies. The higher peptide to lipid ratio utilized in this assay facilitates the occurrence of multiple events including: dilution, membrane partitioning, pore formation, lipid segregation and micellation/solubilisation of the membrane [10]. Each process contributes to the heat-trace and heat of reaction (Fig. 4A–F) with a unique signature. The three latter processes, which are referred to as secondary or reversible processes in this work, are ideally reversible as demonstrated for pore formation in a recent work [10], [37]. For such reversible events, the direction of the process, e.g. pore-formation, lipid segregation or micellation, may revert during the titration sequence. This typically shows as, exothermic heat spikes followed by endothermic heat spikes due to initial formation and subsequent disintegration of a membrane structure e.g. pores. In this study, ITC was used as a screening tool to clarify whether: (i) the peptide analogues interact with POPC∶POPG (3∶1) LUVs via peripheral insertion (partitioning) or (ii) if secondary/reversible processes occur as for the native MPX peptide. In Fig. 4A–C, signatures for secondary/reversible processes are observed for MPX, Leu8, Adec1, Adec14, PAMPX and OAMPX but not for Ala1 and Ala14. For MPX, Leu8, Adec1, Adec14, PAMPX and OAMPX, the signatures are similar to ITC heat-traces previously observed for the pore formation of magainin2, PGLa and MPX [10], [16], [38]. The heat-traces for both Ala analogues (Fig. 4A) are monotonic and resemble the heat-trace for membrane partitioning (as shown in Fig. 3A). The changed behaviour of Ala1 and Ala14 can possibly be explained by the reduced affinity for POPC∶POPG (3∶1) lipid membranes (confirmed by partitioning studies), which decrease the membrane surface density of the Ala analogue and thereby the membrane aggregate formation propensity. However, Leu8, Adec1, Adec14, PAMPX and OAMPX have preserved the MPX ITC signature and may form pores, peptide aggregates or solubilize the POPC∶POPG (3∶1) membrane at these conditions.

The endpoint of the reversible process (highlighted using black arrows in Fig. 4D–F) displays the endpoint sequence MPX∼PAMPX∼Adec1∼Adec14 (L/P∼21)>Leu8 (L/P∼16)>OAMPX (L/P∼11). A large L/P endpoint implies a strongly favoured secondary process as it is difficult to supress by dilution (increasing the L/P ratio). Hence, MPX, PAMPX, Adec1 and Adec14 are more prone to form pores, peptide clusters etc., whereas Leu8 and OAMPX require larger peptide surface densities for these processes to occur. The L/P endpoint obtained for MPX at 37°C is in accordance with our previous finding for MPX at 25°C [10]. For Adec8, the titration was compromised by formation of large aggregates (visual by eye) after the 4'th injection which resulted in a noisy heat-trace. The larger propensity of Adec8 to solubilize the membrane may be related the non-linear molecular structure of this analogue as discussed earlier.

### Bactericidal and haemolytic studies

**Figure 5:**
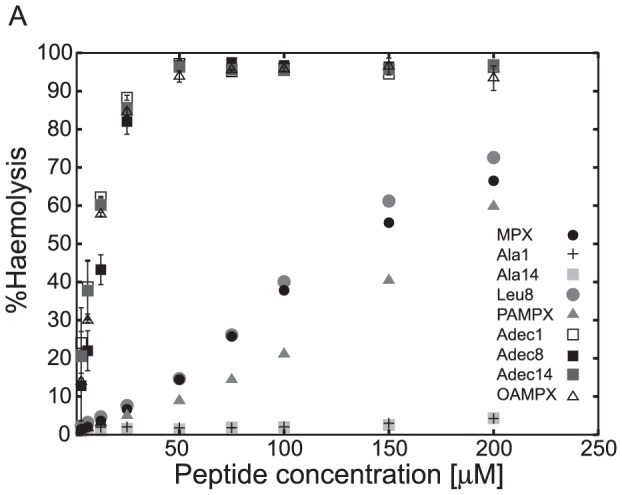
Bactericidal potency of MPX and analogues. The degree of red blood cell haemolysis is plotted as a function of peptide concentration.

Bactericidal and haemolytic studies were conducted, and the results are summarized in Fig. 5 and Table 1. The results are reported as the minimal inhibitory concentrations (MICs) and %haemolysis of human red blood cells (RBCs), which is given as a function of peptide concentration. Adec1, Adec8, Adec14 and OAMPX show the largest potency towards RBCs followed by MPX and Leu8, the latter being slight more potent than MPX at the highest concentrations. PAMPX is found to be less haemolytic than MPX as shown previously [14], while the haemolytic effect of Ala1 and Ala14 is almost completely abolished (∼4% haemolysis at 200 µM). To illustrate this point, full haemolysis is obtained at 50 µM peptide for OAMPX, Adec1, Adec8 and Adec14, while only 15% haemolysis is found for MPX, 10% for PAMPX and ∼1.5% for Ala1 and Ala14. The observed increase in haemolysis for the C8 modified analogues (Adec1, Adec8, Adec14, OAMPX) is in accordance with several reports of C8–C16 acylated peptides, which display increased haemolytic activity upon acylation [24], [26], [39].

The bactericidal potency of Adec1, Adec8, Adec14, OAMPX and Leu8 to *L. lactis* is similar to the potency of MPX, while Adec1, Adec8, Adec14 and OAMPX are ∼2 fold more potent towards *E. coli* than native MPX. Contrary, Ala1 and Ala14 show an 8 fold and 4 fold reduction, respectively, in their potency towards *L. lactis* and *E. coli*, compared to MPX. PAMPX was found to be equally potent towards *L. lactis* as MPX, but 3 fold less potent towards *E. coli*.

**Figure 6:**
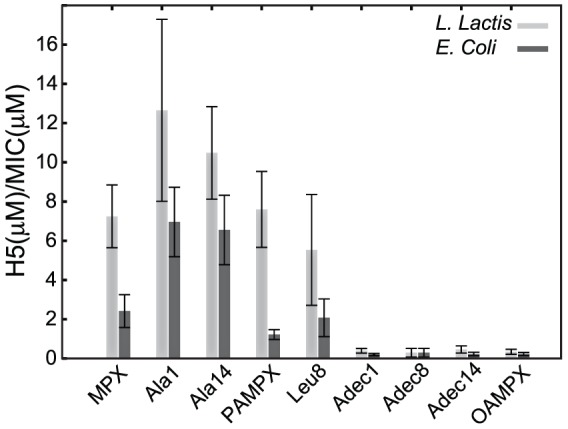
Therapeutic window (membrane selectivity) of MPX and analogues evaluated via the ratio H5/MIC. H5 is the peptide concentration at which 5% haemolysis has occurred and MIC is minimal inhibitory concentration.

In order to evaluate the benefit of the substitution for the eight analogues, their selectivity towards bacteria and RBCs were compared, which is presented as the ratio H5/MIC in Fig. 6. H5 denotes the concentration at which 5% haemolysis has been obtained. As a rule of thumb, H5/MIC>>1 indicate a treatment regime where haemolysis can be avoided at a potent administered dose (peptide concentration>MIC) while H5/MIC<1 implies the opposite; a regime where the peptide targets both bacteria and RBCs. This analysis shows that MPX has a wider treatment window for *L. lactis* compared to *E. coli*. Moreover, Ala1 and Ala14 display an increased H5/MIC value for both *L. lactis* and *E. coli* compared to MPX, while PAMPX and Leu8 show similar selectivity as MPX towards both bacteria strains. Adec1, Adec8, Adec14 and OAMPX show an approximate 15 fold reduction in H5/MIC value compared to MPX, which render these analogues more potent towards RBCs than the tested bacteria strains. Although PAMPX did not show an increased selectivity, as expected from previous work [14], the abolished selectivity of OAMPX correlates with the highly reduced effective charge found previously [14]. The observed reduction in selectivity upon acylation, is furthermore supported by examples in literature [24].

In a recent work, we investigated the secondary structure of MPX, PAMPX and OAMPX using CD (14). In buffer, the analogues predominantly formed beta-sheets and turns (55%), and in the presence of lipid membranes, the analogues were largely structured in alpha-helical segments (50–65%). Overall, MPX, PAMPX and OAMPX exhibited similar behavior respect to structure, and no direct correlation to recent or present results can be established.

The investigated analogues can be divided into 3 groups according to their hydrophobicity: C1 analogues (Ala1 and Ala14) with reduced hydrophobicity, C3-4 analogues (PAMPX and Leu8) with a slightly increased hydrophobicity and C8 analogues (Adec1, Adec8, Adec14 and OAMPX) having the largest increase in hydrophobicity compared to MPX.

The C1 group did not aggregate below 1 mM, and partitions to less readily into neutral lipid membranes (see *K_ins_*, Table 1) compared to MPX. These observations correlate to the almost abolished haemolytic property observed in the RBC assay (Fig. 5A). The C1 analogues showed larger membrane selectivity as measured by the model membrane based *K_eff_/K_ins_* index (Fig. 2C). Similar increase in selectivity was observed for the bactericidal- and haemolytic-based *H5/MIC* index for both of the tested bacterial strains (Fig. 6). For Ala1, *K_eff_* was increased corresponding to enhanced interaction with negatively charged membranes; however, this observation did not translate into an increased bacterial potency for any of the tested bacterial strains (Table 1). Judged by membrane selectivity (*K_eff_/K_ins_*), membrane partitioning (*K_eff_*), and gain in effective charge (*z_eff_*), Ala1 is superior to Ala14, which correlates to the trend (although not significant) observed for the *H5/MIC* index (Fig. 6). Furthermore, ITC heat-traces point to the conclusion, that the mode of membrane interaction is altered as a result of the weaker membrane affinity for both Ala analogues. Alanine substitution (reduction in hydrophobicity) at position 1 compared to position 14 results in a larger increase in effective charge and hence selectivity. This observation may be rationalized by the close proximity of the N-terminal charge in position 1 compared to the larger distance to the neighbouring lysine at position 14 (See Fig. 1).

The C3-4 group did not display aggregation below 1 mM, and the mode of membrane interaction was unaltered as gauged by ITC. Leu8 showed similar bactericidal properties as MPX for both *L. lactis* and *E. coli*. PAMPX exhibited 3-fold lower activity towards E. coli, but equivalent activity as MPX towards *L. lactis*. The C3-4 analogues showed similar potency towards RBCs as MPX, where Leu8 is slightly more potent and PAMPX slightly less potent than MPX. The latter finding is in correspondence with previous observation for PAMPX [14]. Augmented hydrophobicity in position 1 or 8 (see Fig. 1) thus does not impair or boost potency towards bacteria or RBCs, which renders the therapeutic window unaffected.

All C8 class analogues aggregated in the concentration range 10–150 µM, and the cmc's follow the sequence: OAMPX>Adec1>Adec8∼Adec14. Traditional non-ionic surfactants with C8 carbon chain as the hydrophobic moiety have cmc values in the 10 mM range [33]. This is 100–1000 fold higher than cmc of the present analogues, which indicate that the peptide contributes significantly to the overall hydrophobicity of the acylated analogue, and hence to the further reduction of cmc. For all C8 class analogues, the cmc was significantly larger than their MIC and concentration at which full RBC lysis was obtained. Hence, peptide aggregation does not contribute significantly to the biological function of the C8 analogues. Adec1, Adec14 and OAMPX exhibited similar heat-trace profile as MPX (Fig. 4), which indicates unchanged mode of membrane interaction. Adec8, however, showed tendency to aggregate in solution above 125 µM, which caused formation of large aggregates in the ITC experiment when injecting 10 mM LUVs into 100 µM Adec8 solution. All C8 analogues displayed equivalent or slightly increased bactericidal potency compared to MPX; however, their selectivity was significantly reduced causing a radical narrowing of the therapeutic window (Fig. 6).

## Conclusion

Six novel and two known analogues of MPX, having modulated hydrophobicity at position 1, 8 and 14, were investigated using a combined biophysical and biological approach. ITC, bactericidal and haemolytic studies revealed an improved membrane selectivity for alanine substitution at position 1 or 14 of MPX. Leucine substitution at position 8, however, rendered the biological function unaltered. Effects related to the proximity of hydrophobic and charged groups were investigated. This showed that reduction in hydrophobicity close to the N-terminal leads to a larger gain in effective charge and membrane selectivity compared to a reduction of hydrophobicity at position 14; a position more distal to the next lysine. Substitution by 2-amino-decanoic acid at position 1, 8 or 14 or acylation using octanoic acid of MPX rendered these analogues equally or slightly more potent than MPX, however, the membrane selectivity was significantly impaired. Overall these results show that both membrane selectivity and peptide potency can be optimized by acylation/alkylation; still, the present study show that potency is gained at the expense of impaired membrane selectivity or vice versa. Increasing hydrophobicity might improve selectivity in some cases, while in others the selectivity is abolished changing the lipo-peptides' mode of action to a simple detergent-like mechanism that does not distinguish between bacterial and mammalian membranes. The desire for higher potency or membrane selectivity thus determines whether a strategy of increased (alkylation or acylation) or reduced hydrophobicity (alanine or glycine substitution) should be pursued.
